# The Effectiveness of Compression Garments for Reducing Pain in Non-Vascular Ehlers-Danlos Syndromes: A Prospective Observational Cohort Study

**DOI:** 10.3390/healthcare11131862

**Published:** 2023-06-26

**Authors:** Karelle Benistan, Bénédicte Pontier, Catherine Leblond, Ophélie Flageul, Gwenvael Le Guicher, Michel Enjalbert, Fabrice Gillas

**Affiliations:** 1 Reference Center for Ehlers-Danlos Syndromes, Raymond Poincaré Hospital, 92380 Garches, France; 2 Génétique Médicale, Estaing University Hospital, 63100 Clermont-Ferrand, France; bpontier@chu-clermontferrand.fr; 3 Bouffard-Vercelli Center, USSAP, 66000 Perpignan, France; 4 SLBPharma, 35000 Rennes, France; o.flageul@slbpharma.com (O.F.); g.leguicher@slbpharma.com (G.L.G.)

**Keywords:** Ehlers-Danlos syndromes, compression garments, joint hypermobility, pain therapy, orthoses, sprains, dislocation, quality of life, proprioception, joint instability

## Abstract

Patients with Ehlers-Danlos Syndrome (EDS) frequently suffer from severe chronic pain. We carried out an observational cohort study to assess the effectiveness of compression garments (CGs) for reducing this pain. Patients with non-vascular EDS were given custom-made Cerecare^®^ CGs during a visit to a specialist clinic (visit V0). They were followed up over 2 years with visits every 6 months (V1–V4). At each visit, pain was assessed for the joints treated with CGs using a visual analogue scale (VAS; 0–100 mm). Additional measures were obtained to assess neuropathic pain (painDETECT questionnaire), proprioception/balance (Berg Balance Scale), and functional independence, amongst others. Data were analyzed for 67 patients with EDS (hypermobile: 91%; classical: 6%; kyphoscoliotic: 3%). For the most painful joint, the mean VAS rating was 71.5 ± 22.8 mm at V0; this decreased to 53.5 ± 25.5 mm at V1 and 45.7 ± 29 mm at V4 (*t*-tests: *p* < 0.0001). From V0 to V4, improvements were also seen for pain at the other joints, neuropathic pain, functional independence, proprioception/balance, and the incidence of sprains and dislocations/subluxations, although not all comparisons were statistically significant (*p* < 0.05 level). These results indicate that CGs may effectively reduce the pain and joint instability in non-vascular EDS patients.

## 1. Introduction

The Ehlers-Danlos syndromes (EDS) are a heterogeneous group of hereditary diseases that affect the connective tissues. They are characterized by skin hyperextensibility, joint hypermobility, and soft tissue fragility. They are caused by abnormalities in the synthesis or structure of extracellular matrix proteins, particularly collagen, and they affect approximately one in five thousand people [1]. However, accurate prevalence estimates and sociodemographic data are currently lacking [2].

A new classification for EDS was published in 2017, which recognized thirteen different subtypes [3]. Of these, hypermobile EDS (hEDS) is the most common, which represents over 80% of cases [2]. It is characterized by generalized joint hypermobility, which typically leads to recurrent joint dislocations and/or sprains. Nearly all patients also have widespread pain and fatigue [4]. Other EDS subtypes include classical EDS, which is characterized by skin hyperextensibility, atrophic scarring, and joint hypermobility, and kyphoscoliotic EDS; which is characterized by congenital scoliosis, severe muscular hypotonia, and generalized joint hypermobility.

Severe chronic pain is one of the most challenging features of EDS, and it disproportionately affects patients with the hEDS subtype [5]. One study reported that 97% of patients with hEDS were affected, with the pain typically appearing around the age of 10, often following a joint dislocation or sprain. The pain usually becomes chronic by the age of around 20 [4]. It was noted that the pain is often initially localized and then spreads to all joints; there may also be headaches, abdominal pains, and neuropathic pain [4,6,7]. The pain is often debilitating and is associated with psychological distress and a poor quality of life [8]. Many patients have difficulty remaining in full-time education or work, and a substantial proportion suffer from depression [4,9,10].

The mechanisms underlying the chronic pain in EDS are thought to be complex and may involve different factors [11,12]. For instance, the joint instability predisposes patients to recurrent dislocations, soft tissue tears, and overstretching, which give rise to nociceptive pain [13]. There is also evidence for small fiber neuropathy, which was attributed to a reduced density of intraepidermal nerve fibers in these patients [14,15]. Central sensitization is also thought to occur, which may be due to deficits in inhibitory pain control [16,17]. It was hypothesized that patients may have pain-related fear, which leads to avoidance behaviors that may eventually decrease the pain threshold [18]. Other studies focused on proprioception, which helps to protect joints from hyperextension and damage [19] and was found to be impaired in patients with hEDS [20].

As there is no known cure for EDS, treatments are symptomatic. A multidisciplinary approach is usually required, often involving physiotherapy, occupational therapy, orthoses, and complementary therapies (e.g., relaxation training). A variety of approaches can be adopted to treat the chronic pain. Patients may be given analgesics such as non-steroidal anti-inflammatory drugs, paracetamol, or morphine for the nociceptive pain, and drugs such as ketamine, anticonvulsants, and tricyclic antidepressants for the neuropathic pain. However, these are not always well-tolerated by patients and traditional analgesics do not provide adequate pain relief for most patients [12,13]. Other approaches include the use of lidocaine patches, transcutaneous electrical nerve stimulation (TENS), and heat therapy, but again, these may not always provide adequate pain relief [21].

When various therapeutic options were tried and found to be insufficient, compression garments (CGs) were sometimes used [12]. They are usually adopted for patients with hEDS, but they are sometimes also used for those with classical or kyphoscoliotic EDS. The CGs are generally custom-made for patients and are made of elasticated material that applies constant pressure to the skin. They are usually selected to cover specific anatomical regions that may benefit from the CGs, such as the ankles, knees, hips, or hands. It is thought that CGs may be of benefit to patients with EDS by improving proprioception [12,22]. It was suggested that this may involve ‘filtering’ effects, where the CGs reduce the transmission of irrelevant sensory information so that proprioception is improved [23]. It is also possible that CGs may generate input that blocks nociceptive signals before they reach the central nervous system, which would reduce the sensation of pain (gate control theory of pain) [24].

At present, few studies assessed the effects of CGs on pain in patients with non-vascular EDS. In one recent study, Chaléat-Valayer and colleagues [25] assessed the effectiveness of compressive short-sleeved jackets for treating patients with hEDS over a period of 4 weeks. The results showed that there were fewer partial dislocations and dislocations with the CGs, but no significant effect on pain, although the pain rating was found to decrease from 3.5/10 to 2.5/10 (*p* = 0.096).

In 2010, the French National Health Authority (HAS) decided to reimburse the cost of prescription CGs for patients with EDS. However, given the lack of evidence for their effectiveness, the companies supplying the garments were required to set up an observational study to assess their effectiveness over a period of two years, in collaboration with the health authorities. Here, we present the results of this study, which focused on the effectiveness of CGs for reducing pain. It was hypothesized that the CGs would be effective for reducing pain in patients with non-vascular EDS after a period of 6 months. Effects on other measures were also examined over two years, including proprioception/balance, joint instability, fatigue, and functional independence.

## 2. Materials and Methods

### 2.1. Study Design

This study was an observational, prospective, multi-center, non-comparative, descriptive cohort study. The study was registered on clinicaltrials.gov (identifier: NCT03451188) and was carried out in accordance with French law (the ‘loi Jardé’), the Declaration of Helsinki, and the principles of good clinical practice.

### 2.2. Setting

The study was carried out in several specialist centers for EDS, all in France: the Raymond Poincaré Hospital in Garches, the Bouffard-Vercelli Center in Perpignan, and the Estaing University Hospital in Clermont-Ferrand. Patients were recruited for the study between 11 June 2018 and 24 July 2019. Each patient was prescribed CGs, which were received after around one month. The patients were then followed up over a period of two years, up until 30 November 2021, the date of the last study visit.

### 2.3. Participants

Patients were included from the three specialist EDS centers mentioned above. All patients were aged 16 years or older and were diagnosed with EDS, according to the 2017 diagnostic criteria [3], by a physician with expertise in EDS (e.g., orthopedist, dermatologist, rheumatologist, geneticist, internist). There were various inclusion criteria: joint hypermobility, which was inferred by a Beighton score of five or more for patients up to the age of 50, and four or more for patients over the age of 50 [26]; a history of recurrent joint injury (sprains and/or dislocations); not having used CGs for at least 3 months; no anticipated weight changes over the next 6 months (e.g., due to a diet or disease); and having been prescribed Cerecare^®^ CGs. In addition, to be included, patients were asked not to modify their usual care, if possible, such as analgesics, physiotherapy, occupational therapy, or other therapies (e.g., use of TENS, heat therapy, analgesic patches, mobility aids, analgesic medication, flexible or rigid orthoses).

Patients were excluded if they had a known allergy to one of the materials in the CGs; experienced pain resulting from gentle pressure or a light touch (allodynia); experienced acute and unpredictable pains that were not being medically treated at the time of the screening session and would require additional analgesics; were participating in another study; were under guardianship or deprived of liberty; or were pregnant.

Patients were recruited during medical appointments with the clinical investigators. Patients who met the eligibility criteria were provided with oral and written information concerning the study. After a period of reflection, the adult patients provided oral consent to participate in the study; those younger than 18 signed a consent form, and at least one of their parents also signed the form.

### 2.4. Study Timeline

**Figure 1:**
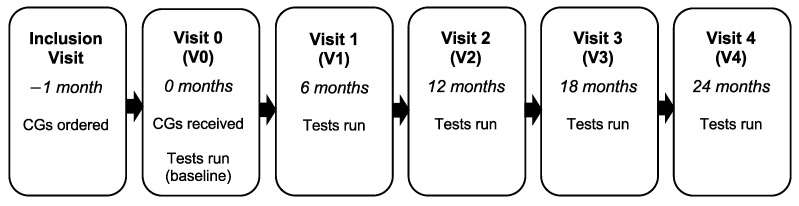
The patients’ visits to the clinic.

Each patient visited the clinic six times as part of the study over a period of two years: an initial inclusion visit, a visit around one month later to receive the CGs (V0), then follow-up visits after 6 months (V1), 12 months (V2), 18 months (V3), and 24 months (V4; Figure 1). For each visit from V0, patients were asked to complete questionnaires and tests to assess their pain, fatigue, joint instability, and quality of life, as well as other measures.

### 2.5. Intervention

Patients were prescribed custom-made Cerecare^®^ CGs (Sailly-lez-Cambrai, France) for their most painful and/or unstable joints. The garments were selected according to the patients’ needs and personal preferences. The patients received two sets of the garments at the medical center after a delay of around one month. Additional sets were given to patients every 6 months, if required. The CGs are similar to those used for severe burns, but they apply less pressure: 10–13.5 mmHg for an area with a perimeter of 24 cm and 6–10 mmHg for an area with a perimeter of 55 cm. The fabric was made of polyamide and elastane, it had >100% elongation for both the warp and the weft (two-way stretch), and it was non-irritant. The CGs included vests with or without long/short sleeves; shorts with or without a long/short extension; gloves/mittens with or without fingers/short sleeves; socks with or without the toes; elbow sleeves; and knee sleeves. For the purposes of this study, treatment compliance was defined as wearing the CGs for at least 6 h per day.

### 2.6. Primary Outcome Measure

The effectiveness of the CGs was assessed by the change in joint pain from V0 (prior to wearing the CGs) for the joint that was judged to be the most painful at V0. Pain was assessed using a visual analogue scale (VAS) from 0 to 10 (0–100 mm; no pain–maximum pain imaginable), where the participants were required to indicate their level of pain on the scale for the month preceding the visit [27]. The main outcome was the change at 6 months (V1), but this was also evaluated after 12 (V2), 18 (V3), and 24 months (V4).

### 2.7. Secondary Outcome Measures

The secondary outcome measures assessed the effectiveness of the CGs for improving various measures over a period of 2 years (up to V4). The following measures were obtained:

- Joint pain for the other joints treated with CGs, as assessed using a VAS from 0–100.
- Neuropathic pain, as assessed using the painDETECT questionnaire (translated into French) [28]. For this questionnaire, participants rate their pain on a numerical scale for three items (0 = none; 10 = maximum pain), they are asked to indicate the pattern of their pain, they are asked to identify the main area where they experience pain, and they are asked to respond to seven questions about neuropathic pain (e.g., burning sensations) using a scale (from ‘never’ to ‘very strongly’).
- Joint instability, as assessed by the number of sprains and dislocations/subluxations for each patient. For this, the patients kept a notebook at home in which they recorded any sprains, dislocations, or partial dislocations from the time of inclusion to the end of the study (V4). As this included the month during which the CGs were being made (first month), the events were compared to this baseline period without CGs.
- Proprioception/balance, as assessed using the Berg Balance Scale [29]. This scale assesses balance control, and so, provides an indirect measure of proprioception. There are 14 different tasks, each rated from 0 to 4 (e.g., standing with eyes closed, standing on one foot), giving total scores from 0 to 56. Scores below 21 indicate that the patient is at high risk of a fall and requires a wheelchair; scores between 21 and 40 indicate that the patient is at medium risk of a fall and requires a walking aid; higher scores indicate that the patient can walk unaided as they are at low risk of a fall.
- Fatigue, as assessed using the Fatigue Severity Scale (FSS; 1–7). This assesses fatigue using a Likert scale for nine different items (1 = strongly disagree; 7 = strongly agree) [30]. If the overall average score is higher than 5.25, the patient is considered to have substantial fatigue, with higher scores indicating higher levels of fatigue.
- Quality of life, as assessed using the Short Form 12 questionnaire (SF-12) [31]. For this questionnaire, participants are asked to select an answer for each of 12 questions, such as whether their health limits them in performing various activities.
- The use of medication or other therapies (e.g., physiotherapy, occupational therapy), as recorded by the clinical investigator at each visit.
- Functional independence, as assessed using the Functional Independence Measure (FIM) [32]. This measure has a total of 18 items (13 motor, 5 cognitive, e.g., eating, problem solving), which each have a score for the level of dependence from 1 to 7 (7 = complete independence). A score greater than 100 indicates independence and the possibility of living at home unaided; a score between 81 and 100 indicates a degree of dependence with some home help required; a score less than 81 indicates dependence, with difficulty remaining at home.
- Incidence of skin problems, as assessed by asking patients the following question: ‘Since your last appointment with your EDS medical specialist, have you noticed any skin changes in terms of bruising, wounds, scars, or stretch marks?’ Patients selected one of the following responses: aggravation, stable, improvement, or not known.
- Patient satisfaction, as assessed using the Quebec User Evaluation of Satisfaction with Assistive Technology (QUEST) questionnaire at V1 and V4 [33]. For this, patients are asked to respond to 12 questions on a scale of 1–5 (‘not satisfied at all’ to ‘very satisfied’). Patients were also asked two additional questions: ‘Did you have any difficulty putting on the Cerecare^®^ compression garments?’ (response: yes/no), and ‘Are you satisfied overall with the Cerecare^®^ compression garments?’ (response: not at all satisfied/somewhat satisfied/satisfied/very satisfied).
- Compliance, which was assessed by asking the patients how many hours the CGs were worn each day (assessed at each visit from V1).

### 2.8. Study Size

A calculation was run to determine the sample size that would be needed to detect a VAS reduction of at least one point (10 mm) after 6 months, with an alpha risk of 5%, a power of 90%, and with 10% of patients lost to follow-up at 6 months. The standard deviation was set at 2, based on the previous literature [34,35,36]. This calculation showed that 76 patients with EDS would be required for this study.

### 2.9. Statistical Methods

Statistical analyses were run using JMP^®^ version 13.0 (Cary, NC, USA). All of the analyses were two-tailed tests with an alpha level of 5%. The statistical analyses were run for the study participants with VAS pain data at V1, irrespective of whether or not they completed the whole study or had other missing data. Additional analyses were also run to support the results, which only included participants who completed the entire study without any major deviations from the protocol.

The data for each study visit were summarized and compared to the baseline values (at V0). Descriptive statistics were presented according to the type of data: means and medians for the quantitative variables along with measures of dispersion (standard deviation and range), and frequencies/percentages for the nominal variables. The values obtained were compared between time points using paired *t*-tests or Wilcoxon tests for the quantitative variables, and McNemar’s tests or Cochran’s Q tests for the nominal variables.

## 3. Results

### 3.1. Participants

**Figure 2:**
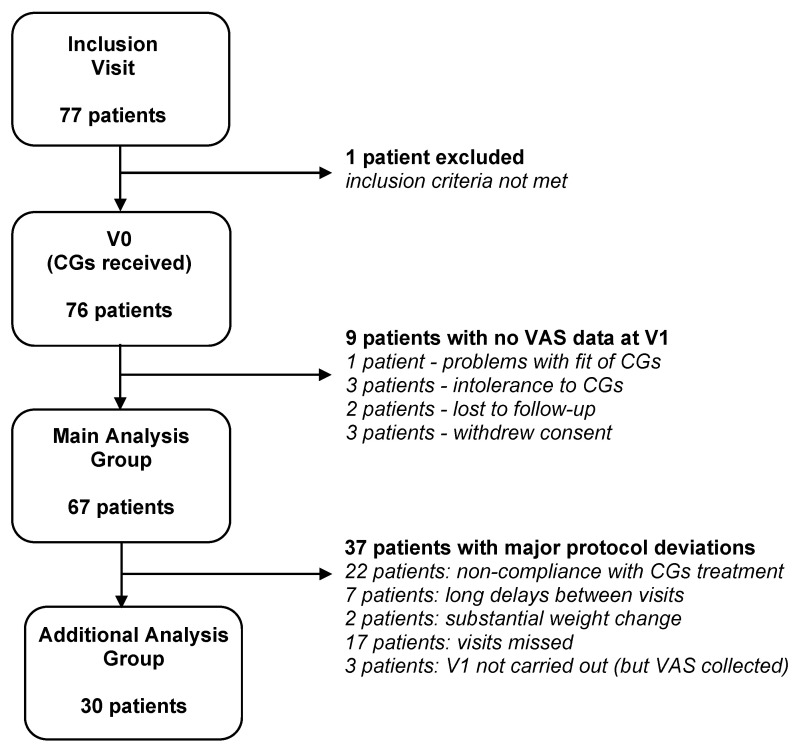
Participant flow diagram. Abbreviations: CGs: compression garments; VAS: visual analogue scale for pain.

A total of 77 patients were recruited for the study. After checking the eligibility criteria, 76 patients were included. Of note, one patient was included who did not fulfill the hypermobility criterion (Beighton score), and another was included who was expected to lose weight. These deviations were accepted by the clinical investigator, as the former patient was diagnosed with EDS by several physicians, and the latter patient was to undergo bariatric surgery, which would be unlikely to have a marked effect on the body areas covered by the CGs (hands, wrists, and elbows). For the main outcome measure (VAS pain at V1), data were available for 67 patients (Figure 2). The main data analyses were, therefore, run using these patients. There were 30 patients who completed the entire study without any major deviations from the protocol; the statistical analyses were repeated using this group to support the study findings. The major deviations included wearing the CGs for less than 6 h per day (non-compliance), long delays between the study visits (>270 days), substantial weight change (>15% body mass index), and missing data for the main outcome measure. Of the remaining patients, a number had minor deviations from the study protocol, which mainly related to the delays between the study visits (beyond the limit of 180 ± 21 days) and the use of online visits. The latter were permitted on occasions (26 visits involving 23 different patients) due to the COVID-19 pandemic, but they meant that the Berg Balance Scale could not be run.

### 3.2. Participant Characteristics

The characteristics of the 67 patients who were included in the main data analyses are shown in Table 1. The patients were aged between 16 and 60 years and they were mostly women (91%). Most of the patients had the hypermobile subtype of EDS (91%) and there were also patients with the classical or kyphoscoliotic subtypes (6% and 3%, respectively). Less than half of the patients previously wore CGs (43.3%). They stopped wearing them for various reasons, including pregnancy, size issues, excessive heat, and difficulty putting the garments on.

### 3.3. Compression Garments and Adherence

**Figure 3:**
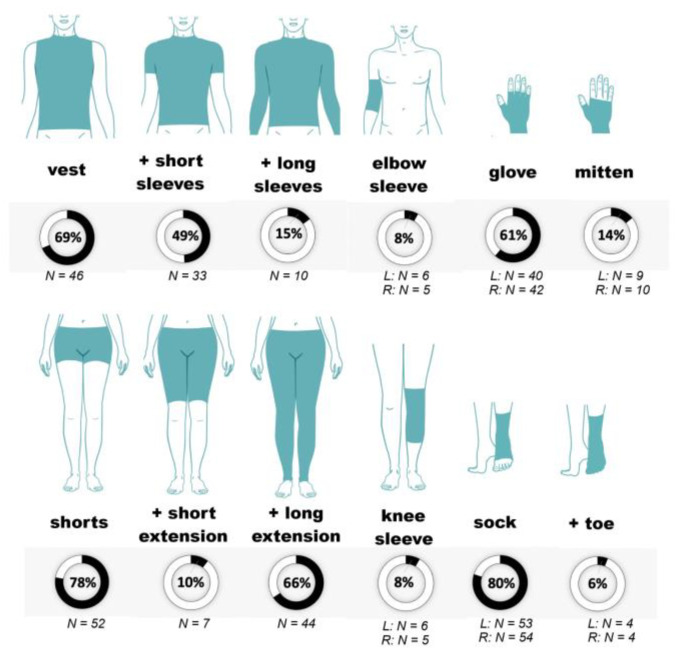
The percentage of patients prescribed each compression garment in the main analysis group (*n* = 67). Means are shown for items that could be for the left or right side (e.g., elbow sleeves), and for gloves and mittens with or without fingers. The garment supplements are indicated by a plus sign. Abbreviations: L: left; R: right.

The patients were prescribed various Cerecare^®^ CGs. These are shown in Figure 3 for the 67 patients included in the main data analyses. The most frequently prescribed CGs were socks (for 80% of the patients), followed by shorts (78%) and vests (69%). The patients were prescribed a mean number of 6.7 ± 1.9 CGs at the inclusion visit (range 2–12).

**Figure 4:**
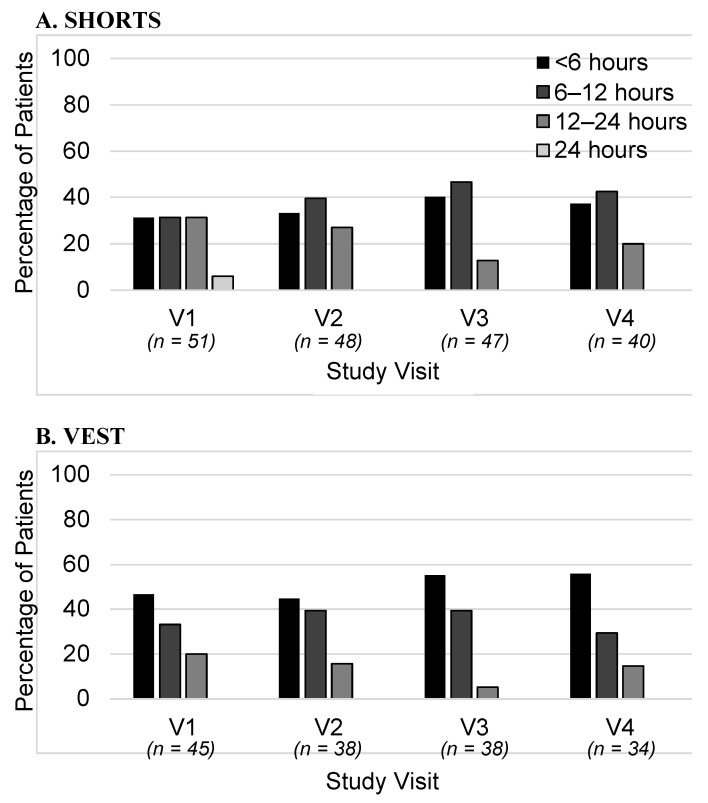
The length of time the compression garments were worn each day. The percentage of patients who wore the garments for <6, 6–12, 12–24, or 24 h is shown, as assessed at visits V1 to V4. Data are shown for shorts and vests.

The approximate length of time each CG was worn per day was recorded at each visit. The durations were grouped into four categories: all day (24 h), 12–24 h, 6–12 h, and <6 h. The percentage of patients who wore the garments for these different durations is shown in Figure 4. Data are shown for visits V1–V4 for the vests and shorts. The great majority of patients wore the CGs for less than 12 h per day, with almost none wearing them for 24 h. There was also a variation between the CGs, for instance, with vests being worn for shorter durations than shorts. The durations did not change markedly over the course of the study.

The patients in the study were followed up for two years. The delays between the study visits were generally well-respected, with a mean delay of 1.2 months (38 ± 12 days; *n* = 76) between the inclusion visit and V0, and mean delays of 6.5 months (197 ± 37 days; *n* = 66), 12.6 months (383 ± 51 days; *n* = 58), 19.2 months (584 ± 70 days; *n* = 55), and 24.9 months (757 ± 59 days; *n* = 50) between V0 and each study visit.

### 3.4. Primary Outcome: Pain Rating at V1

**Figure 5:**
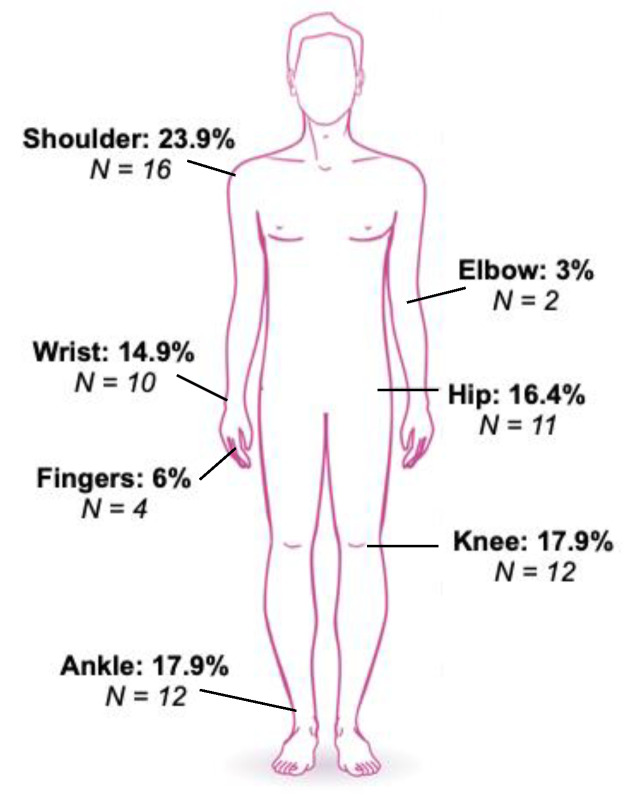
The most painful joint prior to wearing the compression garments. The number of patients who reported each joint to be the most painful is shown, along with the percentages.

The primary outcome measure concerned the joint that was judged to be the most painful prior to wearing the CGs. This was the shoulder for 23.9% (*n* = 16) of the patients, the knee for 17.9% (*n* = 12), the ankle for 17.9% (*n* = 12), the hip for 16.4% (*n* = 11), and the wrist for 14.9% (*n* = 10). A smaller number of patients reported the elbow (3%; *n* = 2) or the fingers (6%; *n* = 4) to be the most painful (Figure 5).

**Figure 6:**
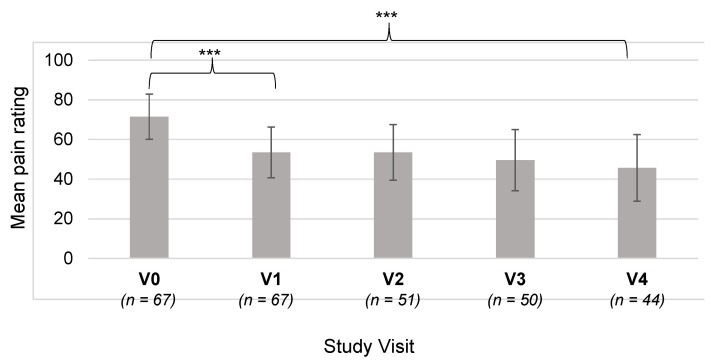
Mean pain ratings over the course of the study for the most painful joint. Pain was assessed using a visual analogue scale from 0–100 mm. The number of patients for each time point is shown along the x axis. *** *p* < 0.0001; error bars: standard deviation.

The mean VAS pain rating was 71.5 ± 22.8 mm for the most painful joint at V0. The pain was assessed again at V1, and the mean rating was found to be 53.5 ± 25.5 mm for the same joint (*n* = 67; Figure 6). This reduction was substantial, at around 25%, and was statistically highly significant (*t*-test, *p* < 0.0001). This shows that the patients’ most painful joint was judged to be less painful after 6 months with the CGs.

### 3.5. Pain Rating at Visits V2–V4

The pain ratings on the VAS for the most painful joint were analyzed for the remaining study visits after V1. The mean values are plotted in Figure 6. The difference between the ratings at V4 and V0 was analyzed to determine whether the pain was significantly lower after 24 months (V4: 45.7 ± 29.0 mm versus V0: 71.5 ± 22.8 mm). It was found that the difference was highly significant (*t*-test, *p* < 0.0001), which indicates that the CGs are effective for reducing pain over relatively long periods of time.

The reduction in joint pain was analyzed for each ‘most painful’ joint separately to determine whether the pain reduction was greater for certain joints. For all of the joints, it was found that the pain was rated to be lower at V4 compared with V0. Statistical analyses showed that the reduction was statistically significant for the ankle, shoulder, knee, and wrist (*p* < 0.05). The differences for the other joints were not statistically significant, but this may be due to a lack of data (Table 2).

### 3.6. Pain Rating at Other Joints

**Figure 7:**
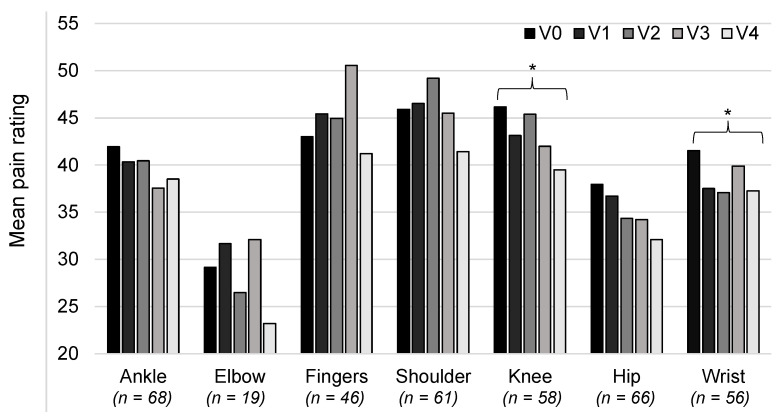
Mean pain ratings over the course of the study for each joint treated with compression garments. Pain was assessed using a visual analogue scale (0–100 mm). The number of patients with data at V4 are shown along the x axis; note that one patient may be counted twice due to data for the right and left sides. * *p* < 0.05.

We ran further analyses to examine changes in pain for all joints that were treated with CGs, not just those that were the most painful at V0. The mean levels of pain for each joint, assessed using the VAS at each visit, are shown in Figure 7. The pain ratings were seen to be lower at V4 compared with V0 for all joints. However, statistical comparison of these two time points showed that the reduction was only statistically significant for the knee (46.2 ± 28.7 versus 39.5 ± 27.3; *t*-test, *p* = 0.015) and the wrist (41.5 ± 28.9 versus 37.2 ± 28.9; *t*-test *p* = 0.03).

### 3.7. Neuropathic Pain

**Figure 8:**
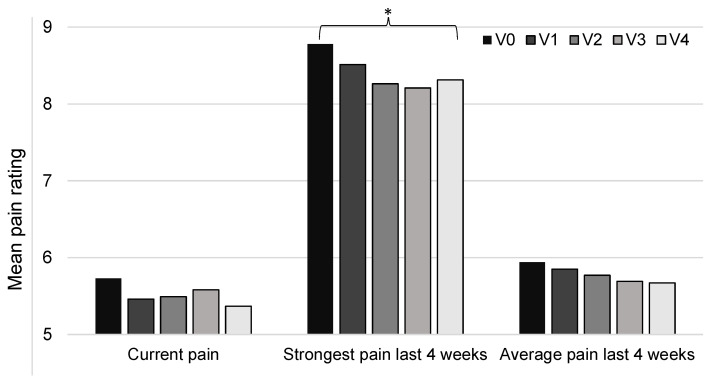
Measures of pain from the painDETECT questionnaire. Pain was rated on a scale of 0–10. There were data for 67 patients at V0 and V1, 57 at V2, 52 at V3, and 49 at V4. * *p* < 0.05.

Neuropathic pain was assessed using the painDETECT questionnaire, which included several measures of pain. The results showed that the mean pain experienced at the time of completing the questionnaire was rated to be 5.7 ± 2.3 at V0 (on a scale of 0–10), and this decreased to 5.4 ± 2.4 at V4. This difference was not statistically significant (*t*-test, *p* = 0.28). Similarly, the rating of the average pain intensity over the last 4 weeks decreased between V0 and V4, from a mean rating of 5.9 ± 1.9 to 5.7 ± 1.5, but again, this was not statistically significant (*t*-test, *p* = 0.32). However, the rating for the strongest pain felt over the last 4 weeks was found to be significantly lower at V4 compared to V0, with mean ratings of 8.3 ± 1.6 and 8.8 ± 1.3, respectively (*t*-test, *p* = 0.048). These results are shown in Figure 8.

The questionnaire also asked patients to indicate the pattern of their pain. The pattern chosen was found to remain relatively stable over the course of the study. It was noted that the most common pattern was persistent pain with pain attacks, which was reported by 64.2% (43/67) of the patients at V0 and 61.2% (30/49) at V4. The next most common pattern was pain attacks with pain between them, reported by 26.9% (18/67) of patients at V0 and 22.4% (11/49) at V4. Few patients reported the other patterns: persistent pain with slight fluctuations (V0: 6%, 4/67; V4: 6.1%, 3/49) and pain attacks without pain between them (V0: 3%, 2/67; V4: 10.2%, 5/49).

**Figure 9:**
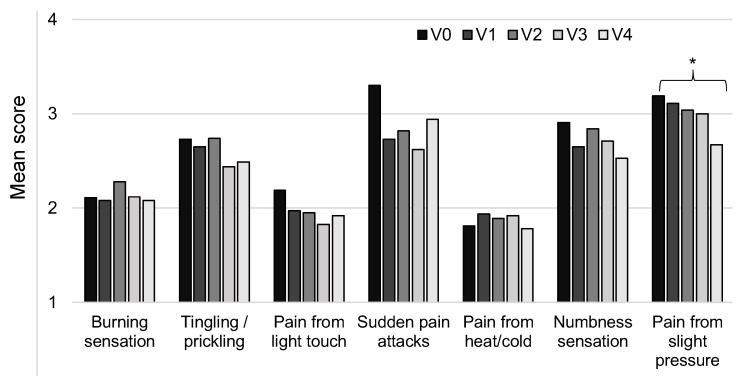
Mean scores for neuropathic pain at the most painful area. Patients responded to items on a scale, and these were converted into a score from 0 to 5. There were data for up to 67 patients at V0 with decreasing numbers down to 49 at V4. * *p* < 0.05.

The painDETECT questionnaire also asked patients to indicate their main area of pain, and they were asked several questions about different sensations and pain responses at this area. The responses were then converted into scores (from 0–5: ‘never’ to ‘very strongly’) and analyzed. The results showed that the measures all improved from V0 to V4, although this was only statistically significant for pain resulting from slight pressure (V0: 3.19 ± 1.58; V4: 2.67 ± 1.56; *t*-test, *p* = 0.049). The results are shown in Figure 9.

### 3.8. Joint Instability

**Figure 10:**
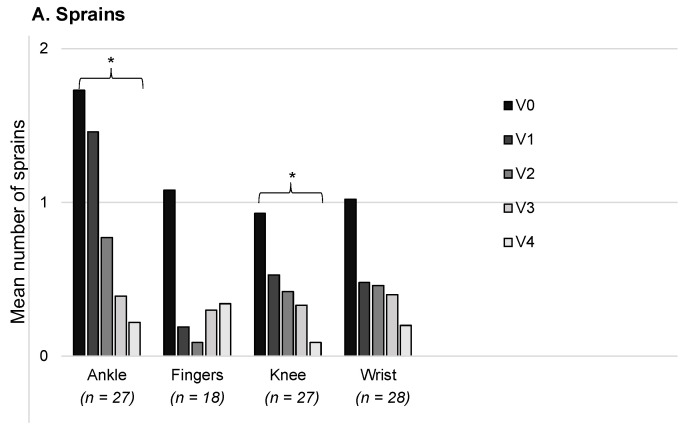

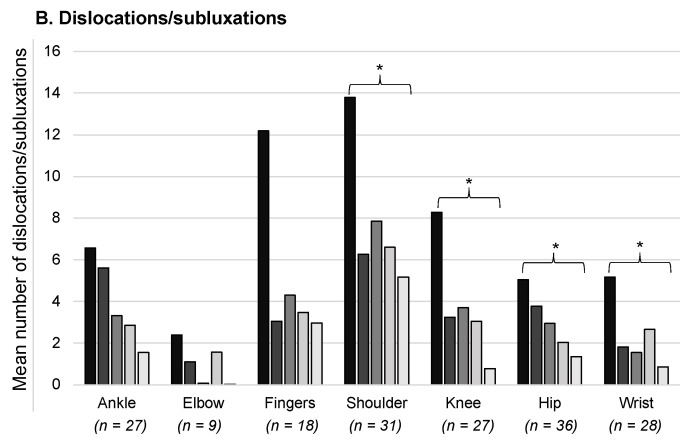
Mean number of sprains and dislocations/subluxations over a 30-day period. The number of patients with data for their joints treated with CG for the duration of study is shown along the x axis. Note that one patient may be counted twice due to data for the right and left sides. * *p* < 0.05.

For each visit, we counted the number of sprains and dislocations/subluxations reported by the patients for the joints with a CG prescription. Analyses were then run, which only included a patient’s data for joints where the CGs were worn from V0 up to V4 for at least 6 h per day. The mean number of sprains is shown in Figure 10A for the ankles, fingers, knees, and wrists; the data are presented for a 30-day period. It was found that there was a decrease from V0 to V4 for all of the joints, although this was only statistically significant for the ankles (V0: 1.7 ± 2.7; V4: 0.2 ± 0.7; Wilcoxon, *p* = 0.0015) and knees (V0: 0.9 ± 2.3; V4: 0.09 ± 0.3; Wilcoxon, *p* = 0.042). The mean number of dislocations/subluxations was also calculated for each joint (Figure 10B), and again, this was found to decrease from V0 to V4. The difference was statistically significant for the shoulders (V0: 13.8 ± 18.4; V4: 5.2 ± 11.9; Wilcoxon, *p* = 0.0012), knees (V0: 8.3 ± 16.6; V4: 0.8 ± 2.9; Wilcoxon, *p* = 0.002), hips (V0: 5.0 ± 9.0; V4: 1.4 ± 3.5; Wilcoxon, *p* = 0.013), and wrists (V0: 5.2 ± 10.9; V4: 0.9 ± 1.9; Wilcoxon, *p* = 0.0018).

### 3.9. Other Secondary Outcome Measures

The patients’ functional independence was assessed using the Functional Independence Measure. The mean score at V0 was 117 ± 8.1 and this increased to 119.8 ± 7.0 at V4. This difference was statistically significant (*t*-test, *p* = 0.001; Table 3). As the mean scores were greater than 100, these results indicate that the patients were mostly able to function independently. The data were examined more closely, and it was noted that most patients were able to walk (V0: 85.1%, 57/67), while a minority walked and used a wheelchair on occasions (V0: 14.9%, 10/67).

We assessed the patients’ balance control using the Berg Balance Scale. The mean total score was 50.2 ± 9.2 at V0 and 51.9 ± 8.1 at V4. This difference was not statistically significant, although there was a tendency towards improved performance (*t*-test, *p* = 0.053; Table 3). As the scores were higher or equal to 41 for 57 of the 67 patients at V0 (85.1%), this indicates that they were at low risk of a fall.

The Fatigue Severity Scale was used to assess the patients’ fatigue. The mean score was 5.8 ± 1.2 at V0 and remained relatively stable, with a mean score of 5.7 ± 1.2 at V4 (*t*-test, *p* = 0.55; Table 3). As the mean scores were above 5.25/7, this indicates that the patients mostly had substantial fatigue throughout the study. Less than 25% of the patients had scores below 5.25 at both V0 and V4, indicating that only a minority of patients had lower levels of fatigue.

The patients’ medication was recorded at each visit. It was found that the patients had two to three drug treatments throughout the study, on average, and that this did not significantly change (*t*-test, *p* = 0.19; Table 3). The different types of medication were also examined, and it was noted that 69.3% (124/179) of the treatments were analgesics (V0). Some patients were also given medication for the gastrointestinal system (12.8%, 23/179) and the nervous system (7.8%, 14/179), amongst others.

The number of additional therapies that patients were receiving was also recorded. The mean was found to be 3.3 ± 1.7 at V0 and 3.3 ± 2.1 at V4 (*t*-test, *p* = 0.80; Table 3). The main therapies were physiotherapy (59.7% at V0, 40/67), heat therapy (58.2% at V0, 39/67), and orthoses (59.7% at V0, 40/67). Some patients also had TENS sessions (43.3% at V0, 29/67), relaxation training (17.9% at V0, 12/67), balneotherapy (14.9% at V0, 10/67), and analgesic patches (38.8% at V0, 26/67), amongst others.

The SF-12 questionnaire was used to assess the patients’ quality of life. In line with the symptoms of EDS, it was found that most patients considered themselves to be in mediocre/poor health (V0: 67.2%; 45/67), had significant difficulty with everyday activities (V0: 58.2%; 39/67), and felt that their emotional or physical state prevented them from doing certain things (V0: 53.7%; 36/67 and 88.1%; 59/67, respectively). They also mainly reported that their pain was very bothersome at work or during activities at home (V0: 73.1%; 49/67). These results remained relatively stable throughout the study.

### 3.10. Patient Satisfaction

Satisfaction with the CGs was assessed at V1 and V4 using the QUEST questionnaire and two additional questions. It was found that most patients were satisfied or very satisfied with the CGs at both V1 (69.8%; 44/63) and V4 (83.7%; 41/49). Only 9.5% (6/63) were not at all satisfied with the CGs at V1 and 0% (0/49) at V4. The responses showed that 68% (34/50) of the patients were satisfied with the effectiveness of the CGs and 52% (26/50) were satisfied with the level of comfort (results at V4). At V1, 52.3% (34/65) of the patients reported not having any difficulty putting the CGs on; this was 57.1% (28/49) at V4.

### 3.11. Effects on Skin and Adverse Events

**Figure 11:**
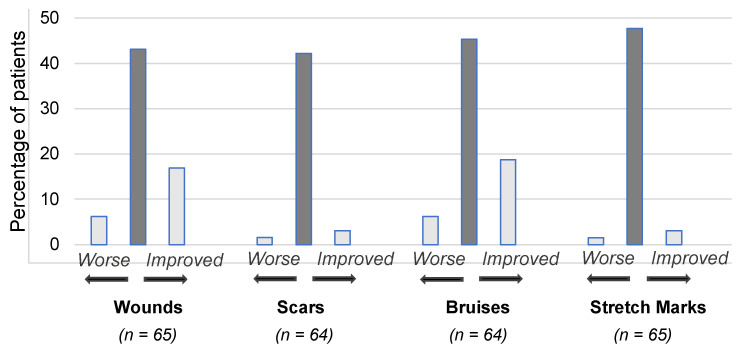
Percentage of patients who reported improvement, worsening, or stability of wounds, scars, bruises, and stretch marks at V1. For each skin condition, the central bar (dark gray) indicates stability, the bar to the right indicates an improvement, and the bar to the left indicates worsening. Note that the percentages do not total 100% because some patients responded that they did not know (not plotted).

At each visit, patients were asked about the condition of their skin relative to their last visit in terms of skin wounds, scars, bruising, and stretch marks. It was found that the skin condition remained relatively stable throughout the study. The results are shown in Figure 11 for V1, when patients were asked to rate changes to their skin since V0 when they did not wear CGs. Most patients reported that there were no changes, but of those who reported a change, a greater number reported an improvement rather than a worsening.

We examined the adverse events (AEs) that were recorded for all 76 patients who received CGs as part of this study. There were a total of 170 AEs, 104 of which related to the CGs, but none of the latter were serious. The AEs included cases of rashes and itching. The AEs led to a temporary discontinuation of the CGs in 59.6% of patients (34/57), and they led to ending the CG treatment in 15.8% of patients (9/57). The AEs that were related to the CGs needed to be treated in 7.6% (13/170) of cases, and in 8.8% (15/170) of cases some other action was required, mainly adjusting the relevant CG.

### 3.12. Additional Analyses

We repeated the statistical analyses using data from the 30 patients who completed the entire study with no major deviations from the protocol. These analyses focused on the results at V4. The results were found to be in line with those presented above and, for certain measures, the improvements were found to be even greater. Importantly, the VAS pain rating was significantly lower at V4 compared with V0 (42.4 ± 28.1 mm versus 69.8 ± 23.4 mm; *p* < 0.0001). For the other comparisons, there were few differences in terms of those that reached statistical significance at the *p* < 0.05 level. The only differences found were in the patterns of significance for the different joints, both for the pain rating (found to be significantly lower for the knee, shoulder, and ankle at V4) and for the number of sprains (significantly lower for the ankle at V4) and dislocations/subluxations (significantly lower for the wrist, knee, and shoulder at V4). In addition, for the measures of neuropathic pain at the most painful area, sudden pain attacks alone were found to be significantly lower at V4.

## 4. Discussion

This study provided evidence that CGs are effective for reducing pain in patients with non-vascular EDS. The improvements were particularly striking for the most painful joint, where the decrease was found to be substantial (around 25%) and statistically highly significant (*p* < 0.001). Reductions in pain were also consistently found for the other joints and for measures of neuropathic pain (from V0 to V4), although not all comparisons were statistically significant. These findings indicate that CGs are appropriate for treating chronic pain in patients with EDS. They may also be effective for preventing recurrent ankle sprains and subluxations/luxations of the wrists, shoulders, knees, and hips, as well as increasing patients’ functional independence.

Most of the patients in this study reported that their pain followed a pattern of persistent pain with pain attacks. Our results suggest that CGs may be particularly effective for reducing the intensity of pain attacks, because significant reductions were found for estimates of the strongest pain over the last 4 weeks. However, as reductions were also found for other measures of pain, this remains to be explored further. Our results also showed that the decreased pain ratings were not short-lived, as they were still seen after two years with the CGs. This indicates that CGs are appropriate for long-term use in patients with EDS.

In our analyses, we considered the different joints separately in order to determine whether CGs are more effective for reducing pain in certain joints. The results suggest that CGs may be particularly effective for treating pain in the knees, wrists, shoulders, and ankles, as significant improvements were found for these joints. However, it is possible that CGs may also be effective for treating pain in the other joints, but that this was not shown due to a lack of data. Further studies are, therefore, required to draw any firm conclusions and to determine which CGs (e.g., vests, gloves, shorts, socks) may be most effective for treating patients’ pain.

As one of the main underlying causes of pain in EDS is thought to be injury resulting from dislocations, subluxations, and sprains [12], we examined the incidence of these. We found that there were consistently fewer sprains and dislocations/subluxations at V4 compared with V0 for all joints, although not all of these were statistically significant. These results were in line with previous studies on patients with EDS, which also found fewer dislocations, subluxations, and sprains when using CGs [25]. Based on these findings, we reasoned that the improvements in pain may at least partially result from a reduction in the number of joint injuries when wearing CGs. If this can be confirmed, it would imply that patients with EDS could benefit from wearing CGs at an early age to avoid joint injury. This could potentially reduce chronic pain over the long term. It may also lead to improved functional independence, as suggested in the present study. However, at present, EDS is typically diagnosed after a considerable delay [4]. For instance, in this study, the patients were diagnosed, on average, 3 years prior to the inclusion visit, despite their mean age of 33 years. This, therefore, underlines the importance of considering the possibility of this disorder in children with joint hypermobility [37], as an early diagnosis could be of much potential benefit.

Previous work suggested that CGs may improve proprioception in patients with EDS and that this may lead to decreased pain [12]. In line with this, several studies showed improved postural control in patients wearing CGs [22,38,39]. In our study, we did not directly test proprioception, but we used the Berg Balance Scale to assess postural control. We found a tendency towards improved performance at V4 compared with V0 (*p* = 0.053). As the patients’ scores were generally high, with 85.1% (57/67) having a score above or equal to 41 at V0, it was concluded that most of the patients did not have deficits in balance control and were at low risk of a fall. To better understand our findings, additional work is required to further explore the potential links between proprioception, joint injury, and pain in EDS.

Our study assessed neuropathic pain using the painDETECT questionnaire. This included questions concerning different sensations and pain responses, such as tingling sensations and pain resulting from slight pressure. The responses to these items showed that most patients experienced these sensations ‘slightly’ or ‘moderately’, thus indicating that they did not have high levels of neuropathic pain. However, for each item, there were patients who selected the maximum response (‘very strongly’), thus indicating that strong neuropathic pain may be limited to certain patients with EDS. This would be in line with a previous study on 31 patients with hEDS, which found small fiber neuropathy in less than two-thirds (61%) of the patients [15]. Our results showed slight improvement in some of the neuropathic pain measures, which indicates that CGs could be effective for improving neuropathic pain in some EDS patients, although this remains to be confirmed in future studies.

When considering the decreased pain ratings in our study for patients wearing CGs, it is relevant to note that the pain levels remained far from negligible, with mean pain ratings of around 40/100 mm on the VAS. This could explain why the patients’ use of medication and other therapies did not decrease over the course of the study, and it could also account for why there was no noticeable effect on their quality of life or levels of fatigue. These findings indicate that CGs should be used alongside other treatments as part of a multidisciplinary approach.

A difficulty with CGs is that they are necessarily tight and may irritate the skin. This is an important consideration for patients with EDS, as the disease is associated with skin fragility and an increased susceptibility to bruising and scarring [3]. In our study, there were cases of itching and rashes resulting from the CGs. However, these were not serious, and in most cases, treatment was not required. We also found that the CGs mostly had no effect on skin bruising, stretch marks, wounds, and scarring, and that the few changes that were reported were mainly improvements. These results, therefore, indicate that the Cerecare^®^ CGs are generally well-adapted to the skin of patients with EDS.

Despite these encouraging findings, some patients in our study reported dissatisfaction with the CGs: 9.5% reported that they were not at all satisfied (at V1). This led to a certain number of patients dropping out of the study, while others completed the study without wearing the CGs as required. Nevertheless, the feedback from patients at the end of the study was generally positive, with over half (52%) reporting satisfaction with the level of comfort and most (57%) having no difficulty putting the garments on. This indicates that the design of the CGs is generally well-suited to patients with EDS, although there is still room for improvement.

This study had several limitations. Firstly, many of the study measures were subjective and, thus, subject to bias, such as the VAS and painDETECT questionnaire. For instance, patients may have expected to have reduced levels of pain with the CGs and this may have led to a bias towards reporting improvements. However, as pain is a subjective sensation, it can be challenging to assess using objective measures [40]. This is particularly the case when considering pain over extended periods of time, as in our primary outcome measure, which assessed pain for the preceding month. Another limitation was that multiple comparisons were carried out without Bonferroni (or other) corrections, which increased the risk of a type I error. However, this did not concern the primary outcome measure (change in pain at 6 months), and the patterns of significance for the other measures were considered with caution. Further difficulties and risks of bias relate to the variability in the treatment with CGs. Specifically, the patients were given different CGs according to their needs, and the patients wore these for variable lengths of time, which could range from just a few hours to 24 h per day; in some cases, the patients did not wear certain CGs at all, and for many patients, the CGs were discontinued for a certain length of time. In addition, for the CGs that were worn, the actual pressure applied to the skin was not verified.

As a final note, this study focused on patients with EDS, but CGs are regularly used to treat patients with burns, lipedema, lymphedema, and orthostatic hypotension. However, these conditions are difficult to compare to EDS. Compression garments are also used to enhance the performance and recovery of athletes [41], who may also suffer from pain, sprains, and dislocations. There is some evidence that reductions in pain may occur in athletes who wear CGs following exercise [42], and many athletes believe that the garments can help prevent sports re-injury [43]. This, therefore, suggests that the benefits seen in this study may apply beyond patients with EDS.

## 5. Conclusions

The results of this study indicate that CGs can be effective for reducing pain in patients with EDS. They may also reduce the incidence of sprains, dislocations, and partial dislocations, and improve patients’ functional independence. However, they may not improve the patients’ fatigue, which can be severe [4], or reduce the need for other therapies and medication. These findings support and complete previous studies on CGs in EDS [25,38,39], and they support the use of CGs for patients with EDS alongside other treatments.

## Figures and Tables

**Table 1:** Demographic and clinical characteristics of the study participants in the main data analyses.

Characteristic	Main Analysis Group (N = 67)
Sex: female, *n* (%); male, *n* (%)	61 (91%); 6 (9%)
Age, years (mean; SD)	33.1 (11.2)
range	16–60
BMI, kg/m^2^ (mean; SD)	25.3 (5.8)
range	16.3–45.7
Activity, *n* (%)	
Employed	29 (43.3%)
Student	15 (22.4%)
No current activity	23 (34.4%)
EDS subtype, *n* (%)	
Hypermobile	61 (91%)
Classical	4 (6%)
Kyphoscoliotic	2 (3%)
Number of years post-diagnosis	2.8 (5.1)
(mean, SD); range	0.01–34.2
Beighton score (mean, SD)	6.3 (1.4)
range	2–9

Abbreviations. BMI: body mass index; CGs: compression garments; EDS: Ehlers-Danlos syndrome; SD: standard deviation.

**Table 2:** Pain reduction at V4 for joints that were the most painful prior to wearing CGs.

Most Painful Joint	Number at V4	Median Pain Rating at V0 (Range)	Median Pain Rating at V4 (Range)	Difference	*p* Value (Wilcoxon)
Shoulder	12	80 (17–100)	60 (20–100)	−20	0.028 *
Elbow	1	68 (66–69)	16 (16–16)	−52	1.0
Wrist	6	78 (50–96)	28 (2–42)	−50	0.036 *
Fingers	2	74 (70–96)	73 (67–79)	−1	1.0
Hip	6	67 (32–100)	38 (18–90)	−29	0.09
Knee	8	67 (30–100)	32 (17–65)	−35	0.016 *
Ankle	9	85 (4–100)	50 (0–98)	−35	0.004 *

* *p* < 0.05.

**Table 3:** Results for various secondary outcome measures.

Secondary Outcome Measure		V0	V1	V2	V3	V4	V4–V0 *p* Value
Functional Independence Measure	Mean (SD)	117.0 (8.1)	118.1 (7.9)	118.8 (7.5)	118.9 (7.1)	119.8 (7.0)	0.001 **
	*n*	67	65	58	55	50	
Berg Balance Scale	Mean (SD)	50.2 (9.2)	51.9 (8.5)	52.8 (5.5)	52.3 (7.5)	51.9 (8.1)	0.053
	*n*	67	63	49	43	47	
Fatigue Severity Scale	Mean (SD)	5.8 (1.2)	5.8 (1.0)	5.8 (1.1)	5.8 (1.3)	5.7 (1.2)	0.55
	*n*	67	65	57	52	49	
Number of additional therapies	Mean (SD)	3.3 (1.7)	3.1 (1.7)	3.2 (1.8)	3.2 (1.9)	3.3 (2.1)	0.80
	*n*	67	65	58	55	50	
Number of drug treatments	Mean (SD)	2.7 (1.9)	2.4 (2.0)	3.0 (2.0)	2.5 (1.7)	2.5 (1.3)	0.19
	*n*	67	67	53	53	49	

Abbreviation. SD: standard deviation. ** *p* < 0.001.

## Data Availability

The data presented in this study are available on request from the corresponding author. The data are not publicly available due to privacy reasoning.
